# Mechano-Electric Feedback in the Fish Heart

**DOI:** 10.1371/journal.pone.0010548

**Published:** 2010-05-07

**Authors:** Simon M. Patrick, Ed White, Holly A. Shiels

**Affiliations:** 1 Faculty of Life Sciences, University of Manchester, Manchester, England; 2 Institute of Membrane and Systems Biology, and Multidisciplinary Cardiovascular Research Centre, University of Leeds, Leeds, England; University of Cincinnati, United States of America

## Abstract

**Background:**

Mechanoelectric feedback (MEF) describes the modulation of electrical activity by mechanical activity. This may occur via the activation of mechanosensitive ion channels (MSCs). MEF has not previously been investigated in fish ventricular tissue even though fish can greatly increase ventricular end diastolic volume during exercise which should therefore provide a powerful mechanical stimulus for MEF.

**Methodology/Principal Finding:**

When the ventricles of extrinsically paced, isolated working trout hearts were dilated by increasing afterload, monophasic action potential (MAP) duration was significantly shortened at 25% repolarisation, unaltered at 50% repolarisation and significantly lengthened at 90% repolarisation. This observation is consistent with the activation of cationic non-selective MSCs (MSC_NS_s). We then cloned the trout ortholog of TRPC1, a candidate MSC_NS_ and confirmed its presence in the trout heart.

**Conclusions/Significance:**

Our results have validated the use of MAP technology for the fish heart and suggest that, in common with amphibians and mammals, MEF operates in fish ventricular myocardium, possibly via the activation of mechanosensitive TRPC1 ion channels.

## Introduction

Mechanoelectric feedback (MEF) is the process by which mechanical forces acting on the myocardium alter its electrical properties [1]–[3]. MEF has been demonstrated in mammalian studies ranging from isolated myocytes to *in situ* human hearts [4]. In addition to the modification of action potential (AP) shape, MEF has been implicated in the generation of arrhythmias (see [5], [6] for reviews).

A mechanism by which MEF may be evoked is the activation of mechanosensitive ion channels (MSCs) [7]–[9]. If channels activated by hyposmotic swelling are excluded, two types of MSCs have been described in cardiac tissue: potassium selective MSCs (MSC_K_) and non-selective cationic MSCs (MSC_NS_) (see [10]). MSC_NS_ have a linear current-voltage relationship with a reversal potential between 0 mV and −30 mV in physiological solutions [11]–[13]. This means that they can potentially cause stretch-induced arrhythmias and early after depolarisations because their activation is predicted to move the membrane potential toward their equilibrium potential [14], [15]. The effect of stretch on the shape of the AP is dependent upon experimental conditions (see [16] for review). However, a specific manifestation of myocardial stretch that is of particular interest with regard to MEF is the ‘cross over effect’. When stretch provokes an initial shortening of the early AP repolarisation time course and an elongation of late repolarisation, it causes a cross over with regard to the stretched and unstretched AP time courses [2], [17]. Such an effect is particularly consistent with the activation of MSC_NS_ that have an equilibrium potential mid-way between the diastolic resting membrane potential and the peak of the AP upstroke [14], [15].

The influence of MSCs on the electrical activity of the intact heart has been studied by measurement of monophasic action potentials (MAPs) [18], [19]. MAPs are extracellularly recorded signals whose time course faithfully reproduces the time course of the intracellular AP [20]. The use of MAP technology has not previously been extended to fish hearts.

Although MSC_NS_ are highly implicated in the modulation of cardiac activity by stretch, their identity is still uncertain. Evidence suggests that transient receptor potential canonical (TRPC) channels are non-specific cationic ion channels that are also mechanosensitive [21] and more specifically that they may be involved in mechanically induced mechanisms in cardiac muscle (e.g. [22], [23]). Recent electrophysiological evidence in adult mammalian ventricular myocytes suggests a role for TRPC1 [24] and TRPC6 [25].

The fish heart is exquisitely sensitive to stretch through the Frank-Starling response which links cardiac output to venous return ([26], [27]). The Frank-Starling response relates the increase in muscle length (that accompanies myocardial stretch) to an increased force of contraction in all vertebrate classes (for review see [28]). Fish are particularly sensitive to this mechanism and can increase stroke volume (SV) by up to 300% during strenuous activity [29]. Consistent with this observation, single isolated piscine cardiomyocytes are highly extensible [30]. The identification and characterisation of MEF in fish hearts is of particular interest given that one aspect of MEF in mammalian tissue is the provoking of arrhythmias [31], while fish hearts can be subjected to sudden and large volume loads without provoking arrhythmias [32].

We tested the hypothesis that MEF is present in fish ventricular myocardium. To do this our first aim was to validate the use of MAP technology in fish myocardium. Next, over a physiological range of input pressures (P_i_s) and output pressures (P_o_s), we investigated the effect of myocardial stretch on the electrical activity of the isolated working fish heart to provide evidence to support the presence and physiological activity of MSC_NS_-like channels. Finally, we confirmed the presence of a candidate MSC_NS_ channel, TRPC1, in the fish heart by cloning the channel and assessing its tissue distribution.

## Materials and Methods

### Fish

All procedures used in these experiments adhere to the local ethics committee and the United Kingdom Home Office Animals Scientific Procedures Act of 1986. The University of Manchester local ethics committee approved schedule 1 killing of rainbow trout with no need for a waiver of consent. Rainbow trout (*Oncorhynchus mykiss*) were purchased from Chirk Trout Farm (Wrexham, UK). The trout were kept in re-circulated freshwater tanks at 11±1°C with a 12∶12 h light-dark photo-cycle and fed with commercial trout pellets to satiation three times a week.

### Isolated whole heart experiments

Fish (430±60 g, n = 8) were sacrificed by concussion of the brain by striking the cranium. The heart (0.65±0.15 g, n = 8) was excised and placed in an organ bath. The heart was then cannulated so that the inflow entered the heart through the sinus venosus and the outflow exited the heart through the bulbus arteriosus. The cannulated heart was perfused with physiological saline which contained (in mM) NaCl, 150; KCl, 5.4; MgSO_4_.7H_2_O, 1.5; glucose, 10; CaCl_2_ 2.5; HEPES, 10; pH of 7.8 with NaOH, oxygenated with 100% O_2_ at 11°C. A tonic level of adrenaline (10 nM) was included in all solutions to preserve cardiac tonus and minimize muscle fatigue [33]. The initial set up procedure did not exceed 8 minutes.

To dilate the ventricle and stretch the myocardium, preload and afterload on the heart could be increased by altering the heights of the reservoirs connected to the sinus venosus and bulbus arteriosus, respectively. Preload was related to P_i_ and afterload to P_o_ by pressure transducers (MLT0380/A, pressure transducer, ADInstruments Ltd, UK) attached to a ML301 bridge pod and Powerlab/4sp data acquisition system (ADInstruments Ltd, UK). Pressure transducers were calibrated daily using a delta-cal transducer simulator/tester (Utah medical, ROI). SV and cardiac outputs were calculated by collecting the solution pumped by the ventricle through the bulbus arteriosus.

Initially the heart was allowed to beat spontaneously to assess the effect of stretch upon heart rate. Cardiac output, heart rate and MAPs were measured as P_i_ was sequentially raised from 0.058±0.003 kPa up to a maximum of 0.322±0.011 kPa. At a P_i_ of 0.322±0.011 kPa, the P_o_ was increased from 0.5 kPa to 7.5 kPa in a stepwise manner. Because AP duration is dependent upon heart rate in the rainbow trout [34], experiments were also performed with extrinsic pacing of the heart by platinum electrodes attached to a SD9 stimulator (Grass, Warwick, USA). A stimulation frequency of 0.8 Hz, using 10 ms square pulses at twice the voltage threshold required for activation, was chosen as this was the lowest frequency at which the intrinsic heart rate was consistently overridden. Additionally, 0.8 Hz is a physiologically relevant cardiac frequency for rainbow trout at 11°C [35].

### MAP recordings in whole hearts

MAP electrodes were made to the design described and validated for use in small mammalian hearts by Knollmann *et al*. [36]. They were constructed from two Teflon coated silver wires (95% purity) 0.25 mm in diameter (Harvard apparatus, Kent), twisted around each other to give the electrodes an inherent spring-like recoil. The distal ends were bent by 90° to each other to produce a recording element and reference element. Electrodes were coated with AgCl, by overnight immersion in bleach, to prevent direct current drift [36]. We also tried larger MAP electrodes built to the design of [37] but found these were less successful at producing stable MAP recordings due to unstable surface contact.

MAPs were recorded from the surface of the ventricle adjacent to the bulbus arteriosus. This was achieved by placing the recording element in contact with the ventricle under slight tension so that the spring-like electrode was able to maintain contact with the ventricle during the filling and emptying stages of the cardiac cycle to eliminate motion artefacts. The reference element was in contact with solution surrounding the heart but not in direct contact with the surface of the heart. Electrodes were connected to an ml136 bio amp and Powerlab/4sp data acquisition system (ADInstruments Ltd, UK). Twelve individual MAPs were recorded and averaged for analysis at each condition tested (see below). MAP duration (MAPD) was assessed at 25%, 50% and 90% repolarisation (MAPD25, MAPD50 and MAPD90, respectively) using Chart v5 (ADInstruments Ltd,). AP triangulation is a measure of the change in shape of the AP in response to an intervention [38]. MAPD triangulation was calculated as MAPD90-MAPD25.

Because this was the first study to measure MAPs in the fish heart, electrocardiograms (ECGs) were simultaneously measured with MAPs to match QT intervals to MAPD as a validation of the duration of ventricular repolarisation. ECGs were recorded from the surface of the heart by electrodes constructed from 2 mm Ag/AgCl pellet electrodes (Harvard apparatus, UK). ECG electrodes were placed on the base and apex of the ventricle [39] and connected to the data acquisition system via a ml136 bio amp (ADInstruments Ltd).

### AP recordings in single myocytes

We also recorded APs from isolated ventricular myocytes from the rainbow trout at 11°C using a whole-cell current clamp to compare the profile of the single myocyte APs with that of the MAP recordings. Myocytes were isolated as previously described [40] using standard intra- and extracellular solutions for AP recordings (see [40] for details).

### Gene cloning

TRPC1 is a candidate cardiac MSC_NS_, it has not been investigated in the trout heart. We cloned the trout variant of TRPC1 (omTRPC1), firstly to determine its tissue distribution, particularly in the heart, and secondly to compare its sequence homology to mammalian TRPC1.

The atrium, ventricle and samples of gill, brain, kidney, intestine, gonad, liver and skeletal muscle were removed from sacrificed trout and immediately frozen in liquid nitrogen. Total RNA was extracted from these tissues with TRIzol reagent (Invitrogen, UK) according to the manufacturer's instructions. RNA was qualified by gel electrophoresis and quantified using an ND-1000 spectrophotometer (NanoDrop Products, Wilmington, USA).

For cDNA synthesis 1 µg total RNA was treated with DNAase I (Invitrogen, Carlsbad, CA) and reverse transcribed with superscript II reverse transcriptase (Invitrogen) and an oligo(dT)_18_ primer, as recommended by the manufacturer. cDNA corresponding to a 350 bp sequence at the 3′ end of omTRPC1 was obtained by PCR using degenerative primers designed to the homologous regions of corresponding mammalian, amphibian, avian and zebrafish genes: (FWD 5′-TGGYTBAGCTAYTTYGATGACAA-3′ and REV 5′-TGGAGGCTTTGGTGKAGAAT-3′). PCR amplification was performed using BIOTAQ^tm^ DNA Polymerase (Bioline, UK), according to manufacturer's instructions, in a 50 µl reaction mixture using a PTC-100 Peltier Thermal Cycler (MJ Research, USA). Amplification was performed under PCR conditions with initial denaturation at 94°C for 2 min followed by 30 cycles at 45°C for 30 s, 56°C for 30 s, and 72°C for 2 min and final extension at 72°C for 2 min. PCR products were checked on a 0.8% agarose gel. 1∶100 dilutions of the PCR products were used in nested PCR reactions with a nested forward primer (5-AAYCATGARGAYAARGARTGGAA-3′) under similar conditions.

The 5′ and 3′ ends of omTRPC1 were amplified using Clontech's SMART RACE cDNA Amplification Kit for rapid amplification of cDNA ends. The cDNAs for the 5′ and 3′ RACE were synthesised from total RNA, from the trout brain, using a modified lock-docking oligo (dT) primer in combination with BD SMART II A oligonucleotide. The 5′ end of the cDNA encoding omTRPC1 was amplified using the omTRPC1 5′Race primer (5′-CAGGTCTCTCATCTCGTTGCGGAACTT-3′) and Universal Primer A Mix (UPM) which recognises the BD SMART sequence. A touchdown PCR protocol was employed following manufacturer's guidelines. A new 5′ primer was designed on the basis of the sequences obtained (5′-ACCTGGAGTGCCGTAGGCTGTCCTTCTTG-3′) and used to clone the remainder of the 5′end under the same conditions. The remaining 3′ end of OmTRPC1 was amplified using a 3′ primer (5′-CCGCTACCTGACCTCCACGCGGCAGAA-3′).

The PCR products were ligated into the pGEM-T vector (Promega, Southampton, UK) and sequencing reactions carried out using Applied Biosystems (ABI, Warrington, UK) BigDye sequencing kit version 1.1. Amino acid sequence comparisons were performed using DNAMAN (Lynnon Biosoft, Canada).

### TRPC1 tissue distribution

Gene specific primers were designed from our sequencing products and β-actin primers were designed using the NCBI nucleotide sequence for rainbow trout β-actin. Reverse transcription polymerase chain reactions (RT-PCRs) were carried out on pooled cDNA prepared from the tissue samples (n = 3). BIOTAQ^tm^ DNA Polymerase (Bioline, UK) was used in 50 µl reaction mixtures, following the PCR protocol: initial denaturation at 94°C for 2 min followed by 25 cycles for β-actin, or 30 cycles for omTRPC1, at 94°C for 30 s, 58°C for 30 s, and 72°C for 2 min and final extension at 72°C for 2 min. Gel electrophoresis on a 0.8% agarose gel was used for qualification and comparison.

### Statistics

Statistical analysis was performed using one-way ANOVA or one-way RM ANOVA followed by the appropriate *post hoc* test, as described in the figure legends (Sigmastat 3.5, Sysstat software, Germany). *p*<0.05 indicated a significant difference. All data are reported as means ± S.E.M.

## Results

### MAP recordings in the isolated fish heart

**Figure 1:**
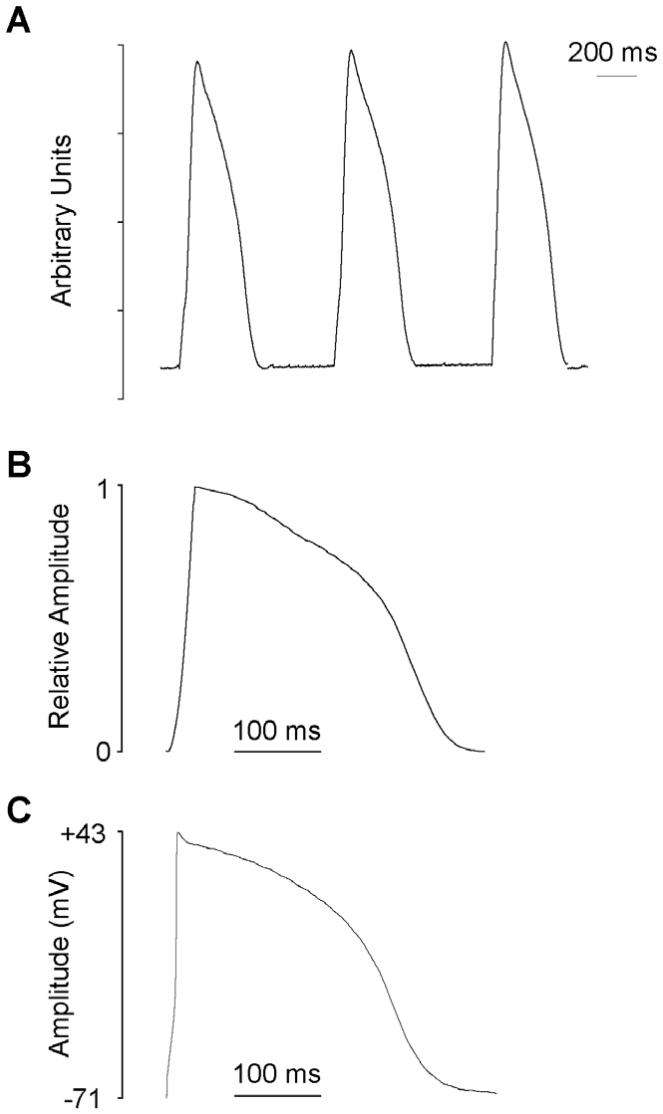
MAPs recorded from fish ventricular myocardium. (A) A train of MAPs recorded from trout ventricular myocardium paced at 0.8 Hz at 11°C. (B) A single MAP taken from the surface of a trout heart spontaneously beating at 0.4 Hz. (C) Current-clamp recording of an AP from an isolated trout ventricular myocyte paced at 0.4 Hz (courtesy of R. Birkedal). The repolarisation time course of the AP and the MAP are similar. The y-axis of the MAP has been scaled to facilitate the comparison of AP/MAP time courses.

**Figure 2:**
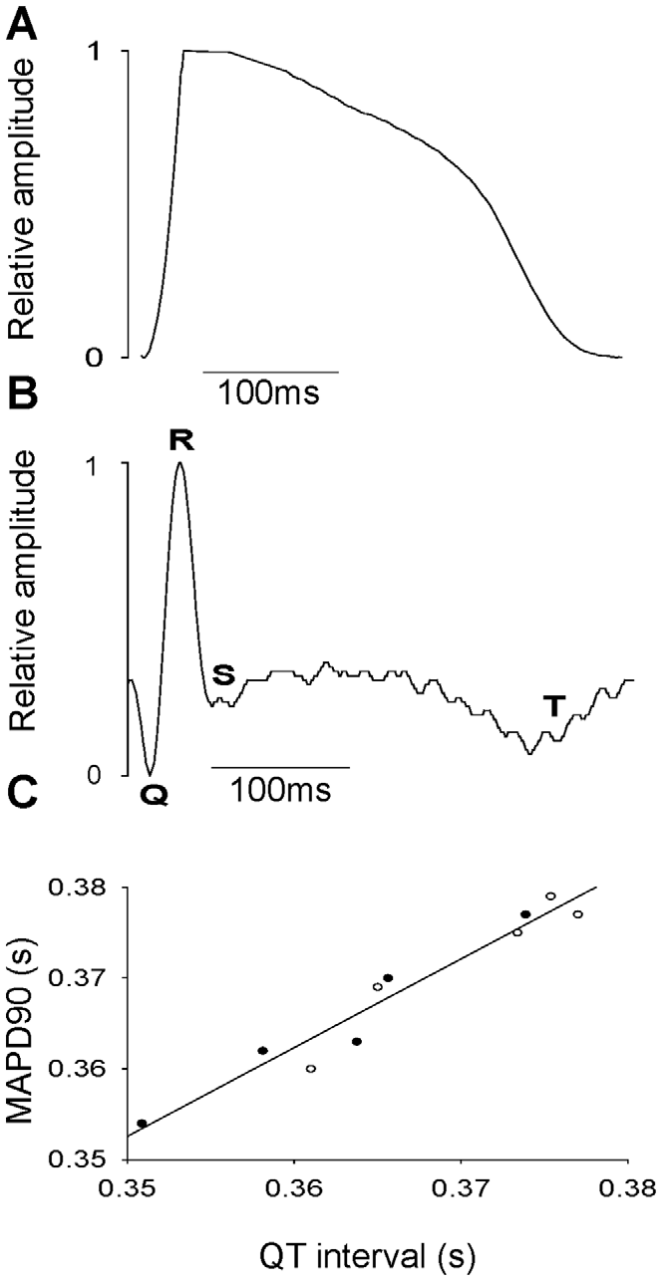
ECGs and MAPs recorded simultaneously from the same fish heart. (A) A MAP recorded from a fish heart extrinsically paced at 0.8 Hz at 11°C. (B) A QT segment from an ECG recorded simultaneously from the same heart. The QRS complex and T-wave are labelled. Note that the QT interval of the ECG trace and the repolarisation time of the MAP are virtually identical. (C) The QT interval of the ECG plotted against average MAPD recorded simultaneously from both spontaneously beating (black circles, n = 5) and paced hearts (open circles n = 5). The gradient of the line is close to unity with a high correlation coefficient (r = 0.97).

We were able to record trains of MAPs from the surface of beating trout hearts that displayed minimal basal line disturbance, indicating negligible motion artefacts in the recordings (Figure 1A). The profile of the trout ventricular MAP (Figure 1B) was consistent with that of previously recorded intracellular APs in the same species (e.g. Figure 1C) the main characteristics being a rapid upstroke, a maintained, elevated phase 2 (plateau) region and an overall duration of several hundred ms. We simultaneously recorded ECGs and MAPs (Figure 2A, B) to provide internal verification that the MAPs faithfully recorded the duration of repolarisation. The QT interval of the ECG (a measure of ventricular repolarisation) was found to be highly correlated with MAPD (Figure 2C); the slope of the relationship was 0.98±0.09. The upstroke times of mammalian MAPs are typically longer than those of intracellular APs and we observed a similar effect in trout hearts (at equivalent temperature and stimulation frequency, 11°C, 0.4 Hz) (see discussion).

### The effect of stretch on the hemodynamics of the isolated fish heart

**Figure 3:**
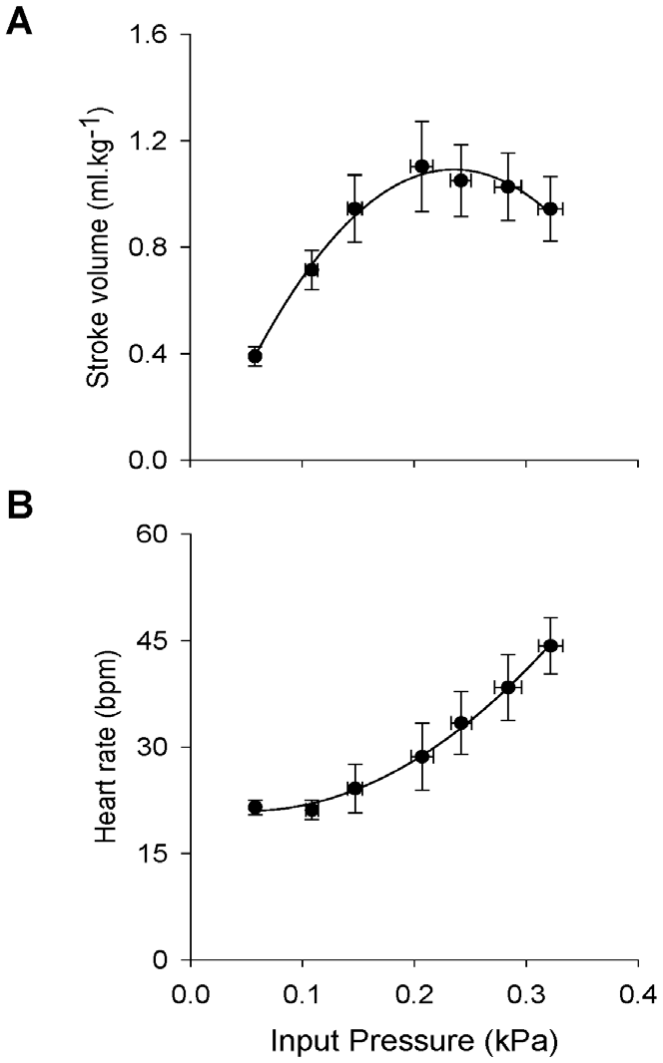
Hemodynamics of the isolated trout working heart. (A) The Frank-Starling relationship in the spontaneously beating isolated heart. SV increases to a maximum (1.10±0.17 ml kg^−1^) at a P_i_ of 0.21±0.12 kPa after which it declines (*p*<0.05, One-Way RM ANOVA, Student-Newman-Keuls *post hoc*, not shown). (B) The increase in heart rate with increasing P_i_ in the spontaneously beating rainbow trout heart with a P_o_ of 0.5 kPa (*p*<0.05, one way RM ANOVA, Student-Newman-Keuls *post hoc*, not shown). Lines represent polynomial fits of the data (all data are means ± S.E.M. of n = 8 hearts at 11°C).

The Frank-Starling response of the spontaneously beating trout heart is shown in Figure 3A. SV significantly increased with P_i_ to a maximum (1.10±0.17 ml kg^−1^) at a P_i_ of 0.21±0.12 kPa (*p*<0.05). In addition, a significant elevation of heart rate from 0.36±0.02 to 0.78±0.07 Hz (*p*<0.05) was observed when P_i_ was increased (Figure 3B).

In the spontaneously beating heart, raising P_o_ from 0.5 to 7.5 kPa at a P_i_ of 0.322±0.011 kPa caused a significant decrease in SV from 0.94±0.12 to 0.60±0.06 ml kg^−1^ (*p*<0.05, not shown). When the heart was paced at 0.8 Hz, increasing P_o_ from 0.5 to 7.5 kPa only reduced SV slightly, by an average of 0.1 ml kg^−1^ (from 0.57±0.09 to 0.48±0.09 ml kg^−1^, not shown), illustrating homeometric regulation of the isolated working heart [41].

### The effect of stretch on the MAP of spontaneously beating hearts

**Figure 4:**
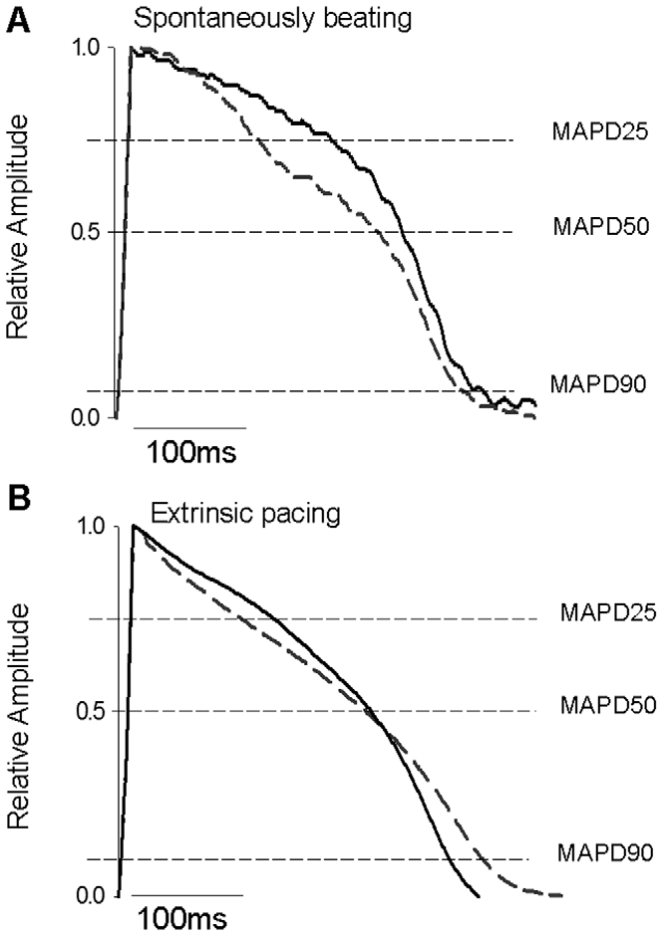
Representative MAP recordings from the working rainbow trout ventricle before and after stretch. The y-axis has been normalised to peak amplitude. (A) MAPs recorded from a spontaneously beating ventricle at a P_i_ of 0.058±0.003 kPa, a P_o_ of 0.5 kPa and a heart rate of 0.35 Hz (solid lines), and with a P_i_ of 0.322±0.011 kPa, a P_o_ of 7.5 kPa and a heart rate of 0.75 Hz (dashed lines). The shortening of MAPD25 can be observed. (B) MAP recordings taken from the surface of a ventricle extrinsically paced at 0.8 Hz. MAPs recorded at a P_i_ of 0.322±0.011 kPa and a P_o_ of 0.5 kPa (solid lines) and 7.5 kPa (dashed lines). A shortening of MAPD25 and an elongation of MAPD90 can be observed showing a classical stretch-induced cross over in the MAP waveform. Recordings shown are the average of 4 traces under each condition.

**Figure 5:**
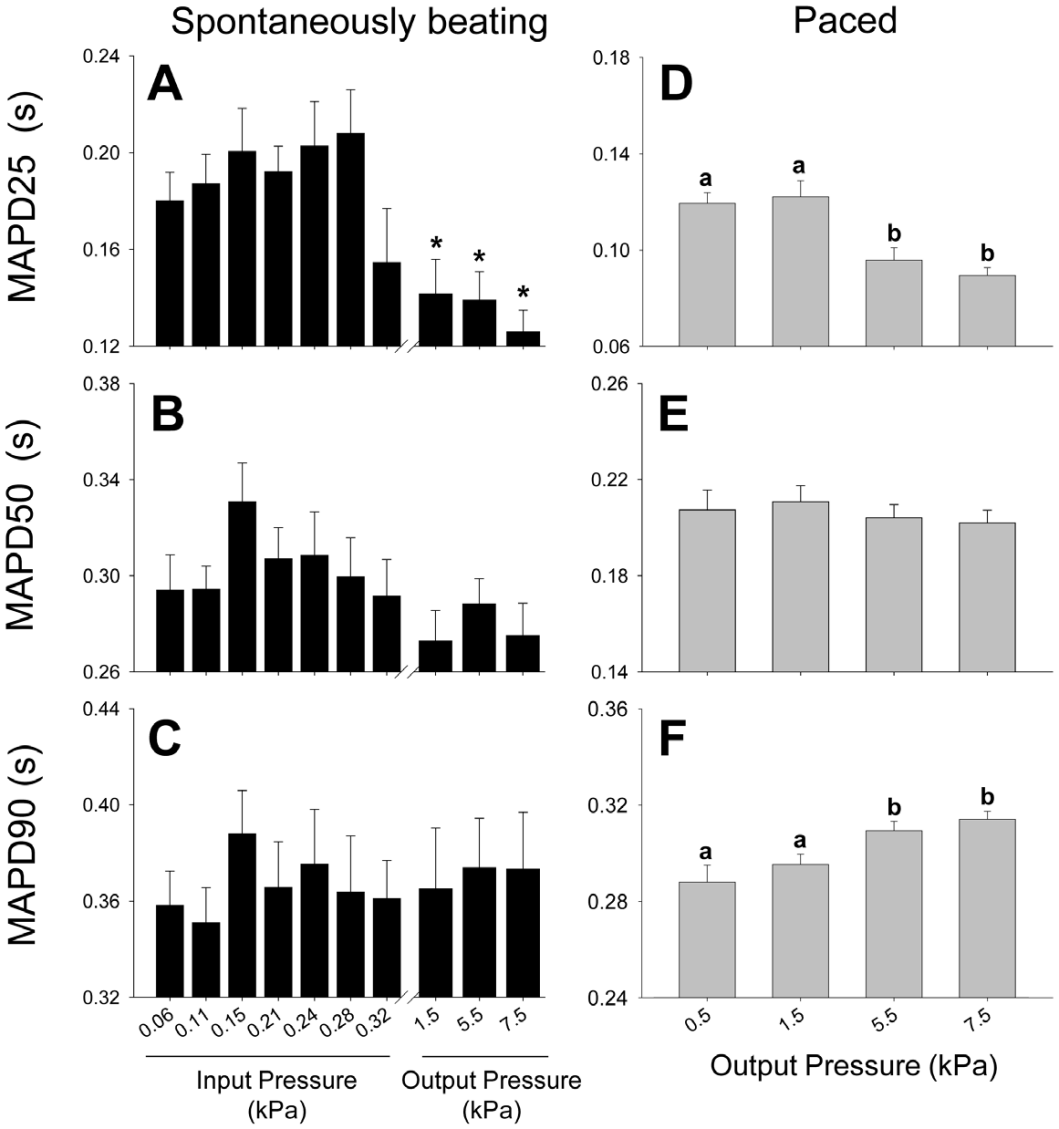
The effect of stretch on the MAP of the fish heart. Spontaneously beating hearts (n = 8) are represented by black bars. These hearts initially had their P_i_ increased to 0.322±0.011 kPa before P_o_ was increased to 7.5 kPa. The grey bars represent hearts extrinsically paced at 0.8 Hz (n = 8). P_i_ for the extrinsically paced hearts was held at 0.322±0.011 kPa and P_o_ was increased. MAPD is shown at MAPD25 (A,D), MAPD50 (B,E) and MAPD90 (C,F) (mean ± S.E.M, *p*<0.05). The only significant effect of stretch upon the MAPD of spontaneously beating hearts is to shorten MAPD25 (* indicate significant differences, *p*<0.05, One-Way ANOVA, Student-Newman-Keuls *post hoc*). Hearts extrinsically paced at 0.8 Hz show a significant decrease in MAPD25 and a significant increase in MAPD90, representing a classical stretch-induced cross over (*p*<0.05, One-Way ANOVA, Student-Newman-Keuls *post hoc*, significant differences between columns marked a and b).

Stretch was applied to the heart via an increase in P_i_ up to 0.322±0.011 kPa, followed by a stepwise increase of P_o_ up to 7.5 kPa. Figure 4A illustrates the effect of stretch on the MAPD25, MAPD50 and MAPD90. Mean data is shown in Figure 5 (A, B, C). When P_i_ was raised to 0.322±0.011 kPa there was a significant shortening of MAPD25 to 155±53 ms (*p*<0.05). Raising P_o_ to 7.5 kPa significantly shortened MAPD25 from all previous conditions (126±21 ms, *p*<0.05) (Figure 5A). Increasing P_i_ or P_o_ had no significant effect on either MAPD50 or MAPD90.

Increased heart rate is known to decrease AP duration in the rainbow trout [34], therefore the changes in MAPD recorded in the spontaneously beating heart might reflect the combined effects of MSC activation and heart rate. To investigate this possibility we repeated the study on isolated hearts extrinsically paced at a fixed stimulation frequency of 0.8 Hz.

### The effect of stretch on the MAP of extrinsically paced hearts

**Figure 6:**
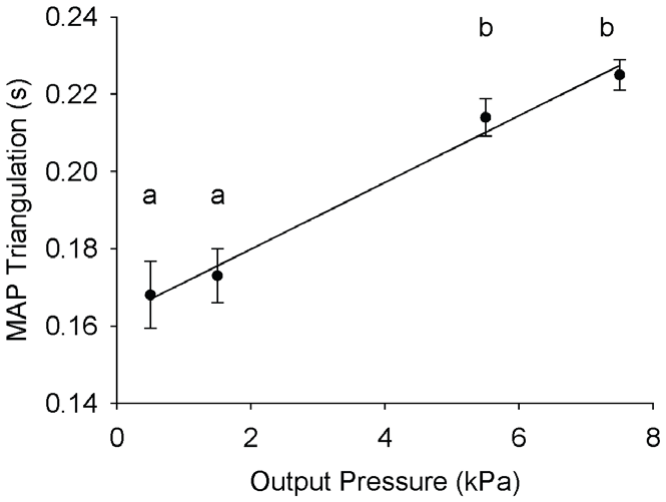
Effect of stretch on MAP triangulation. MAP triangulation is a numerical indicator of AP shape, and was calculated as MAPD90-MAPD25. As P_o_ was increased there was a progressive and significant increase in triangulation as MAPD90 lengthened and MAPD25 shortened. This observation is consistent with graded activation of MSC_NS_ by stretch (*p*<0.05, One-Way ANOVA, Student-Newman-Keuls *post hoc*, significant differences between points marked a and b).

The hearts were paced at 0.8 Hz and P_i_ was held at 0.322±0.011 kPa. Stretch was applied by increasing P_o_ up to a maximum of 7.5 kPa. Figure 4B provides a representative recording which illustrates the effect of stretch on MAPD25, MAPD50 and MAPD90; mean data is given in Figure 5 (D, E, F). MAPD25 was significantly decreased when P_o_ was increased (from 120±4 ms at P_o_ 0.5 kPa to 90±3 ms at P_o_ 7.5 kPa, *p*<0.05). MAPD50 was unaffected by changes in P_o_ (from 207±8 ms at P_o_ 0.5 kPa to 202±5 ms at P_o_ 7.5 kPa, *p*>0.05), whereas MAPD90 was significantly increased (from 288±7 ms at P_o_ 0.5 kPa to 314±3 ms at P_o_ 7.5 kPa, *p*<0.05). The QT interval of the ECG also significantly increased (from 298±3 ms at P_o_ 0.5 kPa to 321±4 ms at P_o_ 7.5 kPa, *p*<0.05; not shown). The effect of these changes on MAP profile can be seen in Figure 4B where a classical stretch-induced cross over between the pre- and post-stretch MAP traces is clearly visible. The progressive change in the shape of the MAP upon increased P_o_ is demonstrated by the significant change in MAP triangulation with increased P_o_ (Figure 6).

### Cloning of trout TRPC1

**Figure 7:**
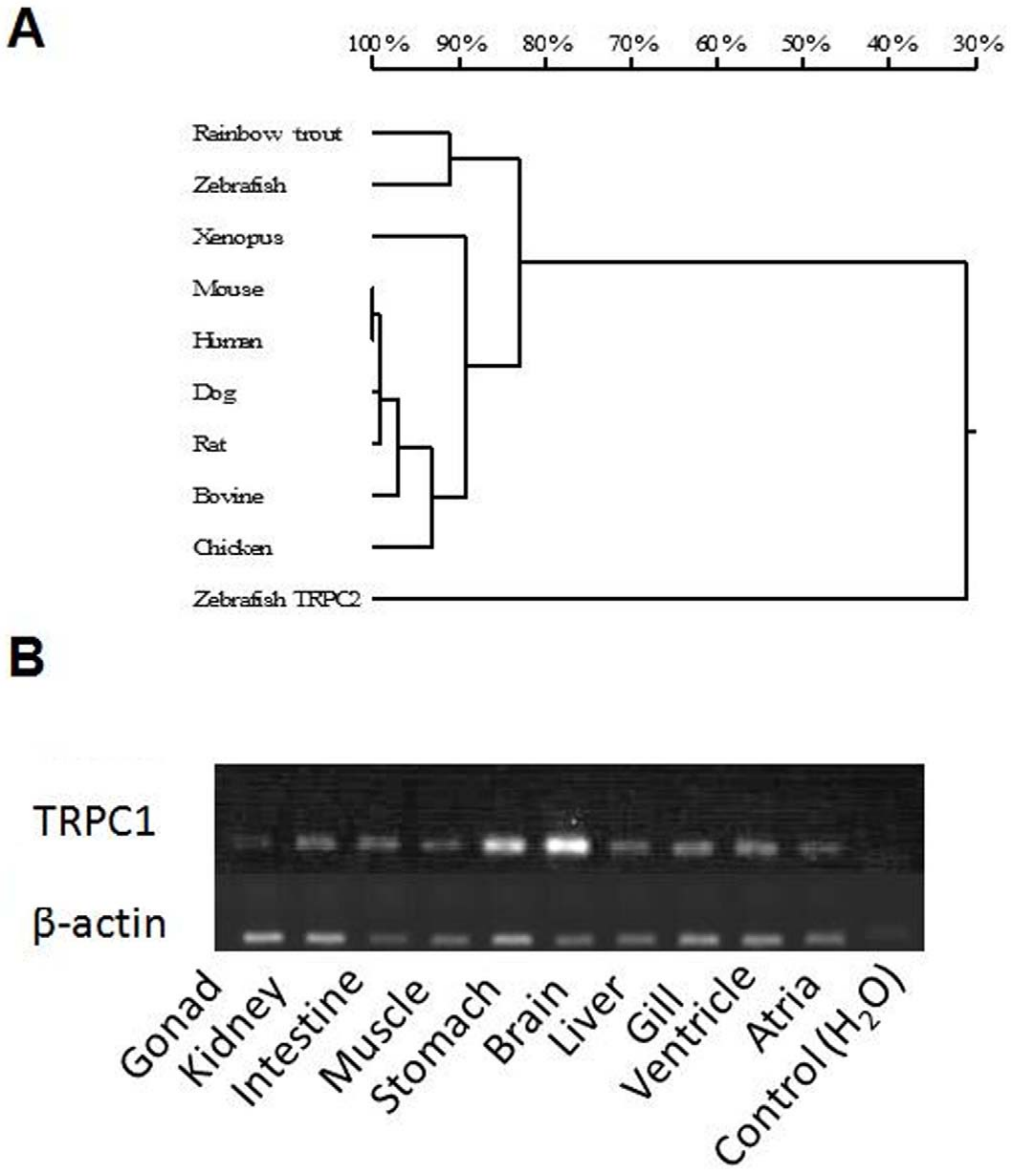
Trout TRPC1 (omTRPC1). (A) Homology tree representative of similarities between the omTRPC1 ortholog and cloned full-length TRPC1 channels of other species. Branch length is percentage homology. (B) Tissue distribution of omTRPC1 and β-actin in various trout tissues.

**Figure 8:**
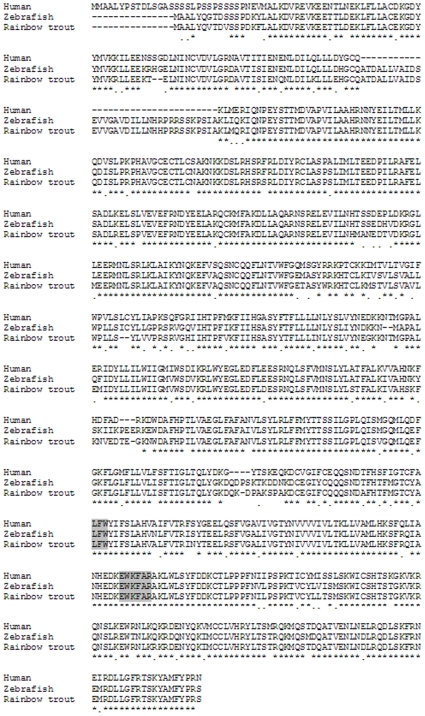
Sequence alignment of omTRPC1. Alignments of the omTRPC1 (accession number: GQ366701) sequence to known homologs in other species using the ClustalW algorithm. Conserved amino acids are denoted by *. The highly conserved channel pore domain (LFW) and TRP box (EWKFAR) are highlighted in gray.

Coding regions of the omTRPC1 gene (accession number: GQ366701) were cloned from rainbow trout cDNA. omTRPC1 is 779 amino acids in length and shares 83.8% homology with human TRPC1 (Figure 7A). This is in contrast to the 30% homology found between omTRPC1 and zebrafish TRPC2, evidence that this gene is indeed TRPC1.The tissue distribution is shown in Figure 7B and confirmed that omTRPC1 is present in both the trout atria and ventricle. It also shows that of the investigated tissues, omTRPC1 is most concentrated in the brain and least concentrated in the gonads. TRPC1 has 6 predicted membrane spanning segments and conservation of amino acids between TRPC1 orthologs is most obvious at the proximal end of the sixth membrane-spanning segment where the highly conserved channel pore domain (LFW) and TRP box (EWKFAR) can be found [42], [43]. Both the channel pore domain and the TRP-box domain are highly conserved in omTRPC1 (Figure 8).

## Discussion

Our MAP recordings are the first from a fish species and exhibit a time course and profile that is consistent with both the QT interval of the associated surface ECG and the intracellular APs recorded from isolated myocytes. The upstroke of the MAP was slower than that of the intracellular AP, which is consistent with previous comparisons of MAPs and intracellular APs from mammals. This is thought to be related to the fact that the MAP is recorded from a small area of tissue around the MAP electrode rather than a single myocyte and that there is a sequential alteration in current flow across this area of tissue [44]. We recommend the use of the small flexible MAP electrodes designed for use in mouse hearts by Knollman *et al*. [36], and conclude that MAP recordings are a valid technique for assessing electrical activity of the fish heart.

The range of P_i_s used in this study are similar to those used in previous work on isolated trout hearts (e.g. [45], [46]) and those used to generate maximum cardiac performance of the trout heart *in situ* [35]. The P_o_s are similar to those recorded in the ventral aorta of trout swimming at 50% maximum prolonged swimming speed [41], [46]. We did not perfuse the coronary system in the current study as previous work has shown that gassing the perfusate with 100% O_2_ ensures ample oxygen supply to working trout myocardium [46]–[48]. Indeed, the stroke work of our preparation (at comparable P_i_s) is equal or greater than that achieved in a similar study where the coronary system was perfused [45]. Moreover, we show a similar increase in SV over a physiological range of P_i_s as seen *in vivo* and *in situ* [46], and SV does not decline rapidly at high input pressures, suggesting our preparation was working over physiological levels of distension [49].

We found that the electrical properties of the isolated trout ventricle were significantly altered when subjected to raised P_i_ and P_o_. We show an initial stretch-induced shortening of MAPD25 in both the spontaneously beating and externally paced isolated heart. This phenomenon was first reported in the frog ventricle [2] and has since been reported in the mammalian ventricle [50], [51]. This initial shortening is thought to be due to the hyperpolarising effect of active MSC_NS_ (and possibly MSC_K_) driving the membrane potential to their respective equilibrium potentials [14], [15].

We saw no change in MAPD90 in the spontaneously beating heart. Stretch increased heart rate and an increase in heart rate leads to a shortening of APD in fish [34] while activation of MSC_NS_ is predicted to lengthen MAPD90. It was therefore possible that our observations in spontaneously beating hearts were an amalgamation of these two effects. In addition to activation of MSCs, stretch may also alter intracellular Ca^2+^ handling and thus Ca^2+^ activated membrane currents [52], [53]. However, when single trout myocytes, contracting auxotonically, were stretched there was no alteration in the intracellular Ca^2+^ transient [30].

At a fixed heart rate of 0.8 Hz, stretch caused a significant shortening of MAPD25 and lengthening of MAPD90. These changes in MAPD25 and MAPD90 are similar to the previously described cross over effect attributed to the activation of MSC_NS_s [14], [17], [44], [51]. If we scale our MAP amplitude to the trout intracellular AP amplitude reported by Harwood *et al*. ([34]), i.e. from 50 mV to −80 mV, our data gives a cross over point in the region of −25 mV. Although this is a rough approximation, it does indicate the cross over within the expected voltage range for MSC_NS_s.

We also observed a stretch-induced increase in heart rate in the spontaneously beating trout heart, this is termed ‘the Bainbridge effect’ [54], and has been observed in a variety of fish species [55], [56]. Although it does not seem to be present in the *in situ* trout heart preparation of Graham and Farrell ([33]), it has been suggested that the Bainbridge effect may be more important in non-mammalian vertebrates which have less neurohormonal control over their hearts [57]. The *in situ* preparation lacks neurohormonal control but the pericardium surrounding the heart is intact. In the present study, we removed the heart from the pericardium so that the MAP electrodes could be placed in contact with the myocardium. Future studies should address the role of the pericardium in MEF in fish.

Having found evidence to support the existence of MSCs in the rainbow trout ventricle, we used molecular methods to investigate the presence of TRPC1 (a candidate MSC) in rainbow trout cardiac tissue. We found omTRPC1 in both the atria and ventricle of the trout heart to be similar to that of most other tissues apart from the brain, where expression was higher. Although mRNA expression does not directly relate to protein expression, the study by Ohba *et al.* [58] shows that expression of TRPC1 mRNA and protein are fairly well matched in the mouse heart. The high expression of omTRPC1 in the brain is to be expected as TRPC1 is involved in processes such as growth cone guidance and the generation of the excitatory postsynaptic potential [59].

The fact that omTRPC1 shares 83.8% homology with mammalian TRPC1 is promising as it may have similar mechanosensitive properties. Unfortunately, it is not known which part of the protein confers mechanosensitivity and so it is impossible, at present, to asses if differences in mechanosensitivity are due to differences in the amino acid sequence.

In terms of the physiological importance of MEF and MSC_NS_ in fish myocardium, the P_o_ required (5.5 kPa) to significantly alter the MAP are comparable to those measured in swimming trout [41] suggesting that MSC_NS_ are of physiological significance when a fish is active. As TRPC1 has been implicated in the promotion of mammalian cardiac hypertrophy [58] it may be implicated in temperature-induced cardiac hypertrophy in fish [33].

This study provides the first MAP recordings from a fish species and is the first investigation into the effects of stretch on the electrical activity of fish ventricular myocardium. There are three principal findings: MAP recordings are a valid technique for use in fish hearts; MEF operates in trout ventricle, within physiological extremes of P_i_ and P_o_, in a manner consistent with the activation of MSC_NS_, and TRPC1 (a candidate MSC_NS_) is present in the rainbow trout heart and is broadly similar to its mammalian ortholog. Thus our data are consistent with the idea that MEF is a common feature in the regulation of vertebrate cardiac muscle. In addition, the T-tubule network has been proffered to explain why, despite recordings of whole cell currents carried by MSC_NS_, no study has successfully recorded single channel activity of MSC_NS_ from mammalian ventricular myocytes [13]. Adult trout ventricular myocytes do not possess a T-tubule network and therefore may provide a useful tool for further study of MSC_NS_ in adult ventricular tissue.
